# Supplementary figures and images for: Adherence to Mediterranean Diet and Biomarkers of Redox Balance and Inflammation in Old Patients Hospitalized in Internal Medicine
Source: Nutrients. 2024 Oct 2;16(19):3359. doi: 10.3390/nu16193359 (PMC11478664; doi:10.3390/nu16193359)

**Supplementary Figure S1.** Diagram flowchart of the study.

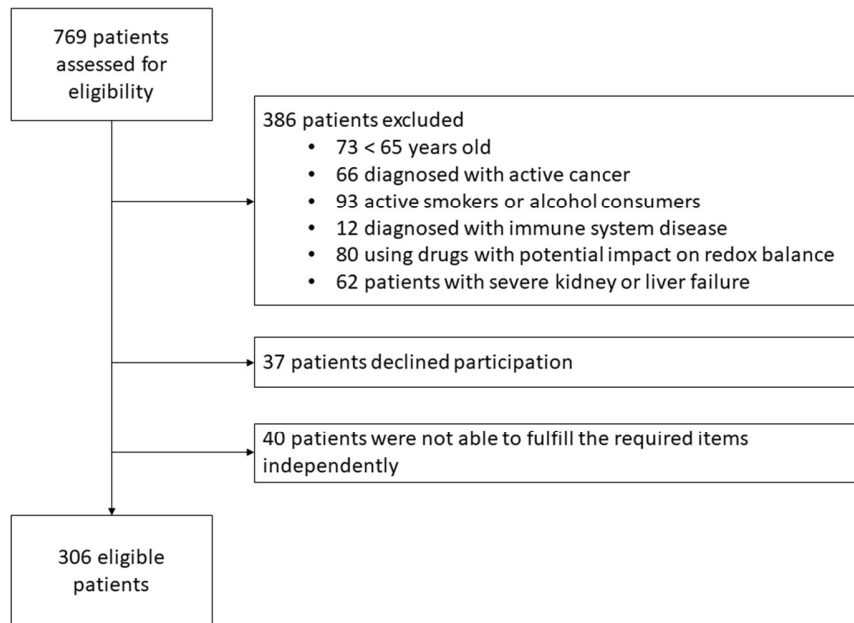

Supplement: Supplementary file 1 [file nutrients-16-03359-s001.zip › nutrients-3209092-supplementary.pdf]
